# Community Acquired Chronic Arthritis due to *Pseudomonas aeruginosa* in a Previously Healthy Pregnant Woman

**DOI:** 10.1155/2014/272306

**Published:** 2014-10-12

**Authors:** Mesut Yilmaz, Ferhat Arslan, Ali Mert

**Affiliations:** School of Medicine, Infectious Diseases and Clinical Microbiology, Istanbul Medipol University, Fatih, 34083 Istanbul, Turkey

## Abstract

Septic arthritis caused by *Pseudomonas aeruginosa* is uncommon in the immunocompetent population, despite its occurrence in younger patients with open injuries and in intravenous drug abusers. Here we report a case of septic arthritis caused by *P. aeruginosa*. This case is unique for several reasons. First, it is a case of septic arthritis in a pregnant woman with no traditional risk factors reported in the literature including history of prior traumatic events, hospitalisation, or chronic underlying disease. She was suspected of having transient osteoporosis associated with pregnancy to involve both hip joints. Second, this is the first reported case of a community acquired chronic septic arthritis due to *P. aeruginosa* involving large joints of both upper and lower extremities. The patient was treated successfully with a combination of ceftazidime and amikacin for 4 weeks followed by oral ciprofloxacin 750 mg twice daily for 8 weeks.

## 1. Introduction

Bacterial septic arthritis is frequently a dangerous and destructive form of acute monoarticular disease and a serious health problem associated with considerable morbidity [1]. Rapid diagnosis, prompt treatment, and adequate therapy are required to prevent serious joint damage. While most cases are limited to the large joints of the lower extremities, involvement of the upper extremity joints rarely occurs [2]. It is caused by a single organism in 95% of cases, and* Staphylococcus aureus* is the most frequent agent responsible for bacterial infections of bone. However, recent reports emphasize the emerging role of Gram-negative bacilli, including* Pseudomonas aeruginosa*, which is the most frequently isolated Gram-negative organism in chronic osteomyelitis. We describe a previously healthy patient who suffered polyarticular arthritis due to* P. aeruginosa* that started during her pregnancy.

## 2. Case Report

A 29-year-old woman presented with a 6-month history of pain with movement of the hips and shoulder joints. She was in the second trimester of her 8th pregnancy when her symptoms started with both hip joints. She had no fever and was suspected of having transient osteoporosis associated with her pregnancy. However, an MRI was not performed at that time and she was managed with symptomatic pain relief. She was reevaluated following delivery as the pain worsened and shoulder joints were also involved. Radiographs and MRI of the hip joints after delivery did not reveal osteopenia but were consistent with septic arthritis. MRI showed effusion, synovial thickening, and alterations in signal intensity of soft tissue and bone marrow of both acetabulofemoral joints.

She was referred to our clinic when bacterial cultures of synovial fluid samples after arthrocentesis of all affected joints revealed* P. aeruginosa*. She did not have diabetes mellitus prior to or during pregnancy and had a BMI of 23 kg/m^2^. She had no history of an immunosuppressive condition, traumatic wound, previous infection, or antibiotic therapy and denied intravenous drug use. She showed no systemic features. A complete blood count was unremarkable but serum biochemistry showed a C-reactive protein level of 100 mg/L and an erythrocyte sedimentation rate of 90 mm/hour. She tested negative for Rose-Bengal test, Wright agglutination tests, and rheumatologic parameters (RF, anti-CCP, ANA, and anti-dsDNA).

Microscopy of synovial fluid showed leukocytosis (40 × 10^9^/L white blood cells) and neutrophilia (88%). Crystals were absent.* P. aeruginosa* isolated from synovial cultures was sensitive to ceftazidime, amikacin, and ciprofloxacin. Blood and urine cultures were negative. The patient received a combination of ceftazidime and amikacin for 4 weeks followed by oral ciprofloxacin 750 mg twice daily for 8 weeks. A full recovery was achieved 3 months later.

## 3. Discussion

Infection can be introduced into a joint either as a result of haematogenous spread or by direct inoculation, occurring with trauma or iatrogenically. Bacteremia is more likely to arise in immunosuppressed individuals and patients admitted to hospital, particularly those who have invasive procedures, intravascular devices, or urinary catheters. Infection will most probably become established if the patient is immunosuppressed or the joint is damaged [3].

Gram-negative organisms may account for up to 20% of cases of septic arthritis [4, 5]. Comorbid medical conditions such as immunosuppression, chronic diseases (alcoholism and diabetes), arthritic joints, intravenous drug abuse, therapy with broad-spectrum antibiotics, and extra-articular infections (particularly urinary infections and decubitus ulcers) predispose patients to Gram-negative septic arthritis [6].* P. aeruginosa* is a rare cause of septic arthritis. Although it is well documented in children and young adults presenting with septic arthritis following a traumatic wound [7, 8] and the elderly [4, 9], to our knowledge our report is the first documented case of septic arthritis in a pregnant woman with nontraditional risk factors reported in English-language literature (Medline 1966–May 2014).

Our patient did not have a history of prior infection during pregnancy or antibiotic use and remained negative for blood and urine cultures on admission; however, we postulate that the joint infections must have followed dissemination of* P. aeruginosa* via the blood from gut following translocation or a urinary tract infection. Negative blood cultures do not refute this hypothesis, since blood cultures are positive in only 62% of cases of septic arthritis [9]. The patient was treated with an antipseudomonal cephalosporin whose synovial fluid concentrations were shown to equate to serum concentrations in animal models [10].

In conclusion,* P. aeruginosa* should be kept in mind as a rare cause of septic arthritis of large joints in the immunocompetent population without classical risk factors.

## References

[1] Kaandorp CJE, Krijnen P, Bernelot Moens HJ, Habbema JDF, van Schaardenburg D (1997). The outcome of bacterial arthritis: a prospective community-based study. *Arthritis and Rheumatism*.

[2] Kelly PJ, Martin WJ, Coventry MB (1970). Bacterial (suppurative) arthritis in the adult. *Journal of Bone and Joint Surgery*.

[3] Mathews CJ, Weston VC, Jones A, Field M, Coakley G (2010). Bacterial septic arthritis in adults. *The Lancet*.

[4] Antón E (2012). Pseudomonas arthritis in an elderly woman. *Journal of Emergency Medicine*.

[5] Goldenberg DL (1998). Septic arthritis. *The Lancet*.

[6] Kaandorp CJE, van Schaardenburg D, Krijnen P, Habbema JDF, van de Laar MAFJ (1995). Risk factors for septic arthritis in patients with joint disease: a prospective study. *Arthritis and Rheumatism*.

[7] Jacobs RF, McCarthy RE, Elser JM (1989). Pseudomonas osteochondritis complicating puncture wounds of the foot in children: a 10-year evaluation. *Journal of Infectious Diseases*.

[8] Yeh TC, Chiu NC, Li WC, Chi H, Lee YJ, Huang FY (2005). Characteristics of primary osteomyelitis among children in a medical center in Taipei, 1984–2002. *Journal of the Formosan Medical Association*.

[9] Keynes SA, Due SL, Paul B (2009). Pseudomonas arthropathy in an older patient. *Age and Ageing*.

[10] Orsini JA, Moate PJ, Engiles J (2004). Cefotaxime kinetics in plasma and synovial fluid following intravenous administration in horses. *Journal of Veterinary Pharmacology and Therapeutics*.

